# Hypercalcemia, Acute Kidney Injury, and Metabolic Alkalosis

**DOI:** 10.1155/2022/1320259

**Published:** 2022-04-06

**Authors:** Faten Aqeel, Jennifer Del Castillo, Bernard G. Jaar, Mohamad Hanouneh

**Affiliations:** ^1^Johns Hopkins University School of Medicine, Department of Medicine, Division of Nephrology, Baltimore, MD, USA; ^2^Department of Internal Medicine, MedStar Health, Baltimore, MD, USA; ^3^Nephrology Center of Maryland, Baltimore, MD, USA; ^4^The Johns Hopkins Bloomberg School of Public Health, Department of Epidemiology, Baltimore, MD, USA; ^5^The Welch Center for Prevention, Epidemiology, and Clinical Research, Baltimore, MD, USA

## Abstract

Calcium regulation is tightly controlled in the body. Multiple causes of hypercalcemia have been studied including primary hyperparathyroidism, hypercalcemia of malignancy, and chronic granulomatous disorders. Among the less studied causes is calcium-alkali syndrome. Here, we discuss a case of hypercalcemia secondary to calcium-alkali syndrome, presenting with hypercalcemia, metabolic alkalosis, and acute kidney injury as a result of ingestion of a large amount of calcium supplements. Hypercalcemia can result in impaired collecting duct system sensitivity to antidiuretic hormone, afferent arteriole constriction, and activation of calcium sensor receptors in multiple tissues. The net effect is an increase in calcium reabsorption with a salt and water diuresis which leads to volume depletion, acute kidney injury, and metabolic alkalosis.

## 1. Introduction

Calcium-alkali syndrome is one of the leading causes of hypercalcemia. It is caused by the ingestion of large amounts of calcium and alkali. The classic triad of calcium-alkali syndrome includes hypercalcemia, metabolic alkalosis, and acute kidney injury (AKI). Here, we report a case of calcium-alkali syndrome secondary to ingestion of calcium carbonate and we review the pathophysiology of this syndrome.

## 2. Case Presentation

A 54-year-old woman with a past medical history of bipolar disorder and gastroesophageal reflux disease (GERD) presented to the emergency room with altered mental status and epigastric pain. Her symptoms were associated with nausea and vomiting for 3 days. Home medications included lithium 300 mg three times a day and quetiapine 100 mg daily. On physical examination, her vital signs revealed a temperature of 99.2°F, heart rate 95 beats per minute, blood pressure 127/72 mm Hg, respiration rate 26 breaths per minute, and oxygen saturation 97% on room air. Pertinent physical findings included soft abdomen with mild epigastric tenderness. She was alert but oriented to person only. Initial laboratory values are listed in Table 1. Electrocardiogram showed a short QT interval (270 ms, QTc 327 ms). Cardiac enzymes were unremarkable. Chest X-ray was normal. CT brain and abdomen without contrast were also unremarkable. Her age-appropriate cancer screening was up to date. Intact parathyroid hormone (i-PTH) level was 13 pg/mL (normal 18–80 pg/mL) which indicated PTH-independent hypercalcemia. Further workup demonstrated a thyroid stimulating hormone (TSH) level of 2.5 *µ*U/mL (normal 0.5–5 *µ*U/mL) and total 25-hydroxy vitamin D level of 11 ng/mL (normal 30–100 ng/mL), excluding hyperthyroidism and vitamin D intoxication, respectively. Parathyroid hormone-related peptide (PTH-rp) was 14 pg/mL (normal 18–80 pg/mL) and total 1,25 dihydroxy vitamin D < 5.0 pg/mL (normal 18–72 pg/mL), making hypercalcemia of malignancy and chronic granulomatous disorder unlikely. In addition, monoclonal gammopathy workup was negative including normal serum and urine electrophoresis with normal serum free light chains ratio. Our patient had the classic triad of calcium-alkali syndrome including hypercalcemia, metabolic alkalosis, and acute kidney injury (AKI). The patient was started emergently on normal saline 300 cc/hour and received IV zoledronic acid 4 mg × 1 dose and calcitonin 4 units/Kg × 1 dose with plans to initiate hemodialysis. However, she responded promptly following the medical therapy (IV fluids, zoledronic acid, and calcitonin) with urine output 200 cc/hour and significant drop in her serum calcium level to 18.3 mg/dL in 1 hour and 15.9 mg/dL in 4 hours. Denosumab was not used due to its lack of availability. Serum calcium level and mental status returned to normal in 24 hours. AKI and metabolic alkalosis resolved in 48 hours. She later reported taking up to 20 tablets of calcium carbonate 500 mg daily for 3 weeks to control her severe GERD symptoms. Given her history of bipolar disorder, her excessive calcium carbonate intake was thought to be related to Münchausen syndrome, prompting a psychiatric evaluation.

## 3. Discussion

The causes of PTH-independent hypercalcemia are summarized in Table 2 [1]. Chronic lithium use can be associated with mild hypercalcemia; however, it is usually PTH dependent (normal to high PTH) [2]. Calcium-alkali syndrome is usually caused by ingestion of large amount of calcium supplements, and it is one of the leading causes of hypercalcemia [3–5]. The classic triad of calcium-alkali syndrome includes hypercalcemia, metabolic alkalosis, and acute kidney injury (AKI). Serum vitamin D is usually suppressed in patients with calcium-alkali syndrome. However, it could be elevated in the case of concomitant excessive intake of vitamin D supplements.

**Figure 1:**
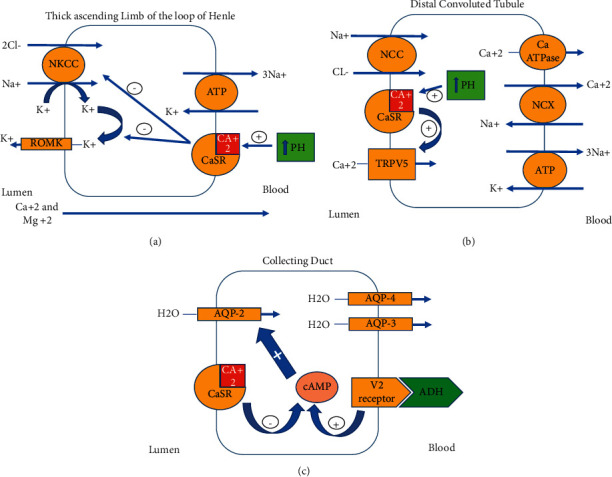
(a) Thick ascending limb of the loop of Henle. (b) Distal convoluted tubule. (c) Collecting duct.

Ingestion of large amounts of calcium leads to an increase in intestinal reabsorption of calcium causing hypercalcemia. Hypercalcemia can result in impaired collecting duct sensitivity to antidiuretic hormone and vasoconstriction of mainly the afferent arteriole in the glomerulus, leading to AKI [5]. Hypercalcemia activates calcium sensor receptors (CaSRs) in multiple tissues. Activation of CaSR in parathyroid gland causes suppression of the parathyroid hormone. Activation of CaSRs on the basolateral membrane of the thick ascending limb of the loop of Henle results in the inhibition of sodium chloride reabsorption and leads to a loop diuretic-like effect and an increase in urinary calcium excretion (Figure 1(a)) [6]. The increase in urinary calcium concentration activates the CaSRs on the luminal membrane of the distal convoluted tubule and the collecting duct cells. Activation of CaSRs on the luminal membrane of the distal convoluted tubules leads to an increase in calcium reabsorption via TRPV5 transporter and worsening hypercalcemia (Figure 1(b)) [6]. Activation of CaSRs on the luminal membrane of the collecting duct reduces the expression of aquaporin 2 water channels, resulting in a decrease in water reabsorption (Figure 1(c)) [6].

The net effect is an increase in calcium reabsorption along with a salt and water diuresis which leads to worsening volume depletion and metabolic alkalosis. Additionally, elevated serum pH perpetuates this cycle by increasing the affinity of CaSRs to calcium. Hypercalcemia causes nausea and vomiting which can also lead to volume depletion and metabolic alkalosis [5].

**Figure 2:**
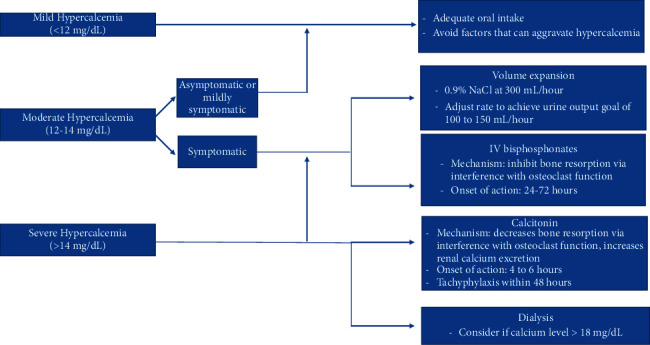
Treatment of hypercalcemia.

The treatment of hypercalcemia is focused on lowering the serum calcium level, while investigating and possibly treating the underlying disease.  Figure 2 summarizes the treatment options of hypercalcemia.

## Figures and Tables

**Table 1:** Initial laboratory data.

Labs	Results	Normal values
White blood count	17.9 K/uL	4–10.8
Neutrophils	80%	
Lymphocytes	8%	
Monocytes	10%	
Hemoglobin	12.8 g/dL	12.5–16.5
Platelet count	327 K/uL	140–400
Sodium	134 mmol/L	135–145
Potassium	3.2 mmol/L	3.5–5.1
Chloride	86 mmol/L	98–107
Sodium bicarbonate	34 mmol/L	22–31
Blood urea nitrogen	50 mg/dL	7–20
Creatinine	4.05 mg/dL (baseline 0.6 mg/dL)	0.7–1.3
Calcium	>20 mg/dL	8.4–10.2
Ionized calcium	3.2 mmol/L	1.17–1.38 mmol/L
Phosphorus	2.6 mg/dL	2.5–4.5
Total protein	7.3 g/dL	6.1–8.2
Albumin	4.7 g/dL	3.5–5.0
Total bilirubin	0.5 mg/dL	0.2–1.2
Aspartate aminotransferase	23 U/L	17–59
Alanine aminotransferase	12 U/L	21–72
Alkaline phosphatase	63 U/L	35–104 U/L
Arterial blood gas		
pH	7.52	7.35–7.45
PCO2	35	35–45 mmHg
PO2	90	75–100 mmHg
HCO3-	32	18–23 mmHg
Urine calcium (mg/dL):urine creatinine (mg/dL) ratio	0.41	<0.14

**Table 2:** Causes of PTH-independent hypercalcemia.

Hypercalcemia mediated by elevated levels of 1,25-dihydroxy vitamin D
** **Granulomatous disorders
** **Foreign material (e.g., silicone)
** **Pneumocystis jirovecii pneumonia
Hypercalcemia from excess calcium intake
** **Calcium-alkali syndrome
** **Calcium sulfate beads
** **Calcium alginate
Hypercalcemia medicated by PTH-related protein
** **Malignancy
** **Benign tumors
** **Sarcoidosis
** **Systemic lupus erythematous
** **Pregnancy/lactation related
Medications
** **Vitamin D and A
** **Thiazides
** **Foscarnet
** **Parenteral nutrition
** **Omeprazole in interstitial nephritis

## Data Availability

Previously reported data were used to support this study and are available at [doi:10.1007/s00268-017-4328-5]. These data are cited at relevant places within the text as references [1]. Previously reported data were used to support this study and are available at [doi:10.1681/ASN.2010030255]. These data are cited at relevant places within the text as references [2]. Previously reported data were used to support this study and are available at [doi: 10.1097/00005792-199503000-00004]. These data are cited at relevant places within the text as references [3]. Previously reported data were used to support this study and are available at [doi:10.2215/CJN.01451005]. These data are cited at relevant places within the text as references [4]. Previously reported data were used to support this study and are available at [doi: 10.1152/ajprenal.00608.2009]. These data are cited at relevant places within the text as references [5].

## References

[1] Motlaghzadeh Y., Bilezikian J. P., Sellmeyer D. E. (2021). Rare causes of hypercalcemia: 2021 update. *The Journal of Clinical Endocrinology & Metabolism*.

[2] Meehan A. D., Udumyan R., Kardell M., Landén M., Järhult J., Wallin G. (2018). Lithium-associated hypercalcemia: pathophysiology, prevalence, management. *World Journal of Surgery*.

[3] Patel A. M., Goldfarb S. (2010). Got calcium? Welcome to the calcium-alkali syndrome. *Journal of the American Society of Nephrology*.

[4] Beall D. P., Scofield H. R. (1995). Milk-alkali syndrome associated with calcium carbonate consumption: report of 7 patients with parathyroid hormone levels and an estimate of prevalence among patients hospitalized with hypercalcemia. *Medicine*.

[5] Felsenfeld A. J., Levine B. S. (2006). Milk alkali syndrome and the dynamics of calcium homeostasis. *Clinical Journal of the American Society of Nephrology*.

[6] Riccardi D., Brown E. M. (2010). Physiology and pathophysiology of the calcium-sensing receptor in the kidney. *American Journal of Physiology-Renal Physiology*.

